# Use of portable blood physiology point-of-care devices for basic and applied research on vertebrates: a review

**DOI:** 10.1093/conphys/cou011

**Published:** 2014-04-04

**Authors:** Lauren J. Stoot, Nicholas A. Cairns, Felicia Cull, Jessica J. Taylor, Jennifer D. Jeffrey, Félix Morin, John W. Mandelman, Timothy D. Clark, Steven J. Cooke

**Affiliations:** 1 Fish Ecology and Conservation Physiology Laboratory, Department of Biology, Carleton University, 1125 Colonel By Drive, Ottawa, Ontario, Canada K1S 5B6; 2 Department of Biology, University of Ottawa, 30 Marie Curie, Ottawa, Ontario, Canada K1N 6N5; 3 John H. Prescott Marine Laboratory, New England Aquarium, Central Wharf, Boston, MA 02110-3399, USA; 4 Australian Institute of Marine Science, PMB 3, Townsville MC, Townsville 4810, Queensland, Australia; 5 Institute of Environmental Science, Carleton University, 1125 Colonel By Drive, Ottawa, Ontario, Canada K1S 5B6

**Keywords:** Biomarkers, field physiology, hand-held blood analyser, non-domestic, validation

## Abstract

Non-human vertebrate blood is commonly collected and assayed for a variety of applications, including veterinary diagnostics and physiological research. Small, often non-lethal samples enable the assessment and monitoring of the physiological state and health of the individual. Traditionally, studies that rely on blood physiology have focused on captive animals or, in studies conducted in remote settings, have required the preservation and transport of samples for later analysis. In either situation, large, laboratory-bound equipment and traditional assays and analytical protocols are required. The use of point-of-care (POC) devices to measure various secondary blood physiological parameters, such as metabolites, blood gases and ions, has become increasingly popular recently, due to immediate results and their portability, which allows the freedom to study organisms in the wild. Here, we review the current uses of POC devices and their applicability to basic and applied studies on a variety of non-domesticated species. We located 79 individual studies that focused on non-domesticated vertebrates, including validation and application of POC tools. Studies focused on a wide spectrum of taxa, including mammals, birds and herptiles, although the majority of studies focused on fish, and typical variables measured included blood glucose, lactate and pH. We found that calibrations for species-specific blood physiology values are necessary, because ranges can vary within and among taxa and are sometimes outside the measurable range of the devices. In addition, although POC devices are portable and robust, most require durable cases, they are seldom waterproof/water-resistant, and factors such as humidity and temperature can affect the performance of the device. Overall, most studies concluded that POC devices are suitable alternatives to traditional laboratory devices and eliminate the need for transport of samples; however, there is a need for greater emphasis on rigorous calibration and validation of these units and appreciation of their limitations.

## Abstract (precis)

Portable blood physiology meters exist that enable researchers to measure various parameters in field settings rather than having to store and transport samples. Although there is need for more thorough calibrations of these devices, they have much promise for conservation physiology of vertebrates.

## Introduction

Blood has been collected from non-human vertebrates for decades to obtain information about organismal physiology, health and condition. Blood is an essential and specialized bodily fluid that delivers necessary nutrients and transports metabolic waste products away from the tissues (Ku, 1997). As a result of its multi-faceted role in supporting organismal life, measurements of the chemical and haematological structure of blood can yield important information, often from a relatively small, non-lethal sample (Rogers and Booth, 2004; Cooke *et al.*, 2005; Wimsatt *et al.*, 2005). Veterinarians and animal health specialists routinely collect blood to evaluate the condition and health of animals (Schalm *et al.*, 1975; Archer and Jeffcott, 1977; Thrall *et al.*, 2012). Today, reference values for a range of species, including domestic and exotic vertebrates, are available to inform veterinary practice and enable health monitoring (see Thrall *et al.*, 2012). Comparative physiologists (see Prosser, 1973) collect and study blood from a variety of model vertebrates in an attempt to understand organismal functions, such as osmoregulation (Beadle, 1957), cardiorespiratory capacity (White, 1978) and the evolutionary basis for, and ecological consequences of, intra- and inter-specific variation (Garland and Adolph, 1991; Garland and Carter, 1994; Spicer and Gaston, 1999). Likewise, much work has been devoted to understanding animal–environment interactions, such as effects of thermal extremes (di Prisco, 1997), living at high altitude (Hall *et al.*, 1936) and seasonal influences on blood physiology (Erickson and Youatt, 1961).

Animal physiologists interested in understanding the consequences of a variety of stressors, such as climate change (Helmuth, 2009) and human disturbance (Busch and Hayward, 2009), also rely on markers in vertebrate blood to identify mechanisms of action, detect thresholds and predict consequences in the growing field of conservation physiology (Wikelski and Cooke, 2006; Cooke and O'Connor, 2010; Cooke *et al.*, 2013). Traditionally, these applications required holding animals in captivity; however, a movement of veterinary practices towards conservation and wildlife surveillance (see Meffe, 1999; Deem *et al.*, 2001) has led to the reinvigoration of comparative physiology, with a focus on the ecological and evolutionary processes in a diversity of non-domesticated taxa (Feder and Block, 1991; Mangum and Hochachka, 1998; Denver *et al.*, 2009; Romero and Wikelski, 2010). This progression has led to the application of physiological approaches needed to understand and solve conservation problems (Cooke and O'Connor, 2010). Fortunately, the ‘field physiology toolbox’ has been expanding rapidly, enabling researchers to study physiological attributes, including blood physiology, outside of the laboratory and even in remote locations (Costa and Sinervo, 2004).

The ability to measure blood physiology repeatedly and accurately has relied historically on large, laboratory-bound equipment and/or complicated and time-consuming assays. While many of these traditional methods are widely accepted and still employed in laboratory/captive settings, they require field biologists to preserve and transport samples for later analysis or to focus on other observable measures, such as behaviour, instead of using physiological measures (St-Louis, 2000). Moreover, certain measurements, such as blood acid–base properties, require immediate reading for maximal accuracy, which is not possible in instances where remotely collected blood is transported back to a laboratory setting. Modern advancements in portable metering or point-of-care (POC) devices have the potential to allow biologists to move towards direct field analysis rather than laboratory-based analysis of samples. Originating in human medicine and later progressing into veterinary science, these devices have shown potential in field biology for a variety of taxa (e.g. Cooke *et al.*, 2008; Atkins *et al.*, 2010; Gallagher *et al.*, 2010; Peiró *et al.*, 2010; Gubala *et al.*, 2012; Sampson *et al.*, 2012).

For field biologists, the usefulness of POC devices is largely tied to their portability, allowing the device to be transported *in situ* with the researcher and study organism for nearly immediate sampling and results (Morgan and Iwama, 1997; Cooke *et al.*, 2008). This early insight provided by POC devices has a range of advantages, including the option to modify a protocol on site, which is useful because pilot testing is often not possible in field studies. In addition, immediate analysis of samples can minimize the potential loss of samples by breakage, transportation and/or degradation (Clark *et al.*, 2011). However, issues may arise when POC devices are applied more broadly, both in terms of environmental conditions and taxonomic group (Costa and Sinervo, 2004). To be functional broadly in field environments, POC devices must compensate for fluctuations in background humidity and temperature as well as account for the physiological differences between relatively stable homeotherms (for which most POC devices are initially calibrated) and heterothermic models that vary along with their external conditions (Costa and Sinervo, 2004; Gallagher *et al.*, 2010; Mecozzi *et al.*, 2010). Physiological conditions and species-specific differences may further complicate the reliability of POC devices, leading to inaccurate and/or imprecise results; as such, the development of species-specific blood physiology ranges may be necessary. Although there are obvious drawbacks to the use of POC devices in the field on non-domesticated and less studied organisms, the ease of use and portability of these devices may largely outweigh the disadvantages. In addition, owing to the affordability of POC devices, the growing number of validation studies on non-domesticated species and the continuing technological advances (e.g. more parameters, greater portability), these devices are likely to play an integral role in modern biology for basic and applied studies.

In this review, we have collected and synthesized information from a number of studies that have used portable POC devices to measure blood physiology parameters from non-domesticated vertebrate animals. Although POC devices vary in size, our review was limited to ‘easily portable’ POC devices due to their increased applicability to field biology. We defined ‘easily portable’ as any device <5 kg that was powered by a self-contained battery and did not require external electricity or compressed gas canisters. We assumed, based on those criteria, that devices could be carried easily by backpack, horseback, small aeroplane, canoe, etc. to remote areas. The objectives of this review were as follows: (i) to examine the current uses of POC devices in medical and veterinary science; (ii) to summarize the calibration and application of POC devices for use on non-domesticated vertebrate taxa (fish, reptiles, birds and mammals); (iii) to identify the limitations of such devices; and (iv) to consider potential future uses of POC devices.

## History of point-of-care device development

In the past two decades, POC devices have revolutionized at-home patient care and emergency diagnostic capability by enabling paramedics, healthcare professionals and even patients to measure biochemical markers rapidly and accurately. The use of POC devices in humans was first recorded in 1994, and by 2009 25% of tests were being performed at the site of care (Plebani, 2009). Based on their success and convenience, the use of POC devices has been projected to increase by 12% per year (Plebani, 2009).

These devices can and have been used in various settings, including management and treatment of disease symptoms in the home and in paramedic care (i.e. ambulance), as well as use in field diagnostics (Plebani, 2009; Cima, 2011). Perhaps the most widespread application of such POC devices is for diabetic monitoring of blood glucose, with many variants available for purchase from pharmacies, which have been integrated into day-to-day management of insulin levels by millions of people around the world (Klonoff, 2005). Furthermore, POC devices have been integrated into emergency departments; for example, some units can measure biochemical markers for patients with acute chest pains suggestive of myocardial injury to provide a rapid, whole-blood analysis in 20 min (Apple *et al.*, 2000). Ideally, these devices should help to reduce turnover time for patients in hospitals and, by doing so, enable better utilization of resources, reduce time to discharge and ensure better patient management (Altinier *et al.*, 2001). Not only will the technology of POC devices reduce healthcare costs in North America but, owing to the portability of these devices, it also has the potential to allow the distribution of this healthcare across the world and into less developed regions (Cima, 2011). Human uses have been expanded further to include the monitoring of performance athletes (e.g. lactate levels) and, indeed, some POC devices have been developed for that explicit purpose (e.g. Lactate Pro; Pyne *et al.*, 2000).

Since the introduction of POC devices in the medical field, veterinary science has adopted their use in clinics to monitor the health of animals (Allen and Holm, 2008). As a result of their origins in human medicine (Acierno *et al.*, 2007), quality control is necessary because of the many physiological and biochemical differences across taxa (Allen and Holm, 2008); however, there are a growing number of such devices designed specifically for domesticated animals (e.g. Gluco Pet). Testing the precision of a POC device, or validation, is done by comparing animal- and parameter-specific values with those obtained from a ‘gold standard’ bench-top analyser or laboratory assays (e.g. Clark *et al.*, 2008). The process of validation of POC devices is ongoing, as new devices and biomarkers used to assess animal health are constantly emerging. This task has proved to be even more complex because a specific range of measured values for a particular parameter may vary in its concordance with a bench-top analyser (e.g. POC values may agree better with laboratory-based values inside but not outside of a specific range; Hollis *et al.*, 2008).

Once validated for a certain animal-specific physiological parameter, POC devices have been shown to improve the efficiency of establishing both a diagnosis and a prognosis for that animal (Acierno *et al.*, 2007). Point-of-care devices allow for easy and early identification of sick animals and do not require specialized laboratory personnel (Steinmetz *et al.*, 2007). For example, blood lactate as a measure of tissue hypoxia in sick animals can be measured quickly and accurately with a POC device using a small volume of blood (Mizock and Falk, 1992). Due to the low cost associated with the use of POC testing, many private animal owners, in particular horse owners, use these devices for establishing treatment action, because this early information can be used to determine whether additional, more expensive testing or treatment is necessary (Delesalle *et al.*, 2007). In addition, as new biomarkers are discovered, the hope is that new POC devices will also emerge, benefitting not only veterinary medicine, but also other fields, such as conservation and field physiology.

## General approach

We examined various studies that used POC devices on a range of non-domesticated vertebrate taxa. Despite the prominent use and origins of POC devices in emergency patient care and veterinary medicine of domesticated animals (e.g. horses, dogs, cats, mice), we excluded all such studies herein, given our focus on wild, traditionally non-domesticated animals. In some cases, it was unclear whether animals (mostly fish; e.g. Gomes *et al.*, 2006a, b; Trushenski *et al.*, 2010) in captivity were wild or captive bred, so we relied on our best judgement. We did encounter several studies that used POC devices with invertebrates (e.g. Allender *et al.*, 2010; Butcher *et al.*, 2012), but focused solely on vertebrates, given that invertebrate blood physiology is less well studied. As noted above, we included only studies that used devices considered to be ‘easily portable’. In addition, we excluded all portable devices that were not electronic (e.g. refractometers for plasma protein) or that simply separated blood constituents [e.g. manual or electronic centrifuges for quantifying haematocrit (Hct)].

Primary literature searches were conducted between 15 September and 18 November 2012. Relevant papers were found by searching a variety of academic journal databases (e.g. Web of Science, Scopus) and Internet search engines (e.g. Google Scholar) with relevant search terms (e.g. ‘point-of-care analyser’, ‘portable clinical analyser’, ‘glucose meter’), as well as identifying citations by other papers. To identify papers that fitted our criteria, we reviewed the study design to determine whether a POC device was used. We then extracted key information and populated a database with predetermined headings, such as species, parameters tested, POC device used, type of study and limits. Studies were classified either as validations, which compared the POC devices with traditional laboratory analysis, or as applications, which used POC devices to measure blood chemistry for either basic or applied research. In some studies, POC devices were used for both validation and application purposes, and such studies were therefore classified accordingly as both.

## Characteristics and trends in point-of-care device literature

### General characteristics

The literature search yielded 79 studies involving the use of POC devices in a variety of non-domesticated vertebrate species. The use of POC devices in ecology and conservation is a relatively novel concept, because the majority of published articles have appeared within the last decade and range from 1995 to the present. We collected articles from various journals (*n* = 42), which included a wide variety of themes, such as veterinary medicine, disease, toxicology, conservation, physiology and food science. Of the 79 peer-reviewed studies, 8.9% (*n* = 7) of articles were published in *Fisheries Research*, while 7.6% (*n* = 6) of articles were published in *Comparative Biochemistry and Physiology Part A*. Validation studies comprised 17.7% (*n* = 14) of papers, from which applications studies accounted for 78.5% (*n* = 62) of the total. Only 3.8% (*n* = 3) of studies combined both validation and application.

### Taxonomic patterns

We retrieved papers from four different taxonomic vertebrate groups, namely fish, birds, reptiles and mammals. Due to the large number of fish studies and the associated physiological differences between teleost and cartilaginous fishes, we separated all *Chondrichthyes*-based studies into a separate group. Teleost fish were the most-studied taxa, accounting for more than half of the total number of articles (*n* = 48; 60.8%). Studies focusing on *Chondrichthyes* accounted for 15.2% (*n* = 12) of all studies, while mammals, reptiles and birds accounted for 11.4% (*n* = 9), 8.9% (*n* = 7) and 3.8% (*n* = 3), respectively. Atlantic salmon (*Salmo salar*) was the most-studied species (8.9%; *n* = 7), while studies focused on smallmouth bass (*Micropterus dolomieu*) and Atlantic cod (*Gadus morhua)* were the second and third most-studied organisms, with 7.6% (*n* = 6) and 6.3% (*n* = 5), respectively.

### Point-of-care device tools and parameters tested

In total, studies used 20 different POC devices that fit the ‘easily portable’ definition (see Introduction). Although we found several studies using the VetScan analyser, which is considered a POC device by the authors of those papers, it did not fit our definition of ‘easily portable’ and was therefore excluded. The i-STAT hand-held blood analyser was the device most used (53.2%; *n* = 42), followed by the Lactate Pro lactate meter and Accu-chek glucometer, which were used in 27.8% (*n* = 22) and 24.1% (*n* = 19) of assessed studies, respectively (Table 1). In addition, glucometers were the most diverse POC device, with nine different models being used. The majority of devices used (85%; *n* = 17) measured only one parameter, such as the Lactate Pro, while the i-STAT analyser was the most used device that was capable of analysing more than one blood parameter. Finally, the majority of studies used whole blood, rather than plasma, when using the POC devices (94.9%; *n* = 75).

**Table 1:** List of most common point-of-care devices used in studies analysed (note that not all data are available due to products being discontinued)

Device	Company	Parameters tested	Additional cartridges/strips	Type of blood required	Amount of blood required (μl)	Battery required	Range (mmol/l unless otherwise stated)	Dimensions (length × width × height; mm)	Weight (g)	Current validations	Temperature range (°C)	Humidity range (relative humidity unless stated)
Accu-chek Advantage	Roche Diagnostics/Boehringer Mannheim	Glucose	Yes; Accu-chuk strips	Whole	0.6	One 3 V lithium battery	0.6–33.3	84 × 53 × 21	60	No	10–40	10–90%
IQ Prestige	Home Diagnostics Inc.	Glucose	Yes; Prestige IQ strips	Whole	4	One AAA 1.5 V alkaline battery	1.4–33.3	70 × 102 × 20	102	Burdick *et al.* (2012), mammal	15–37	Any non-condensing atmosphere
Ascensia Elite	Bayer Corporation	Glucose	Yes; Ascensia Elite Test strips	Whole	2	One 3 V lithium battery	1.1–33.3	81 × 51 × 14	50	No	10–40	20–80%
ExacTech	Abbott Point of Care	Glucose	Yes; ExacTech strips	Whole	10	Not available	Not available	93 × 55 × 10	43	No	Not available	Not available
Freestyle Freedom Lite	Abbott Point of Care	Glucose	Yes; Freestyle Freedom Lite strips	Whole	0.3	One 3 V lithium battery	1.1–27.9	8.38 × 5.08 × 1.3	42.35	Wells and Pankhurst (1999), fish	4–40	5–90% non-condensing
Glucometer Elite	Bayer Corporation	Glucose	Yes; Glucometer Elite strips	Whole	2	Two 3 V lithium batteries	1.1–33.3	97.8 × 56 × 14.5	60	No	10–40	20–80%
Sure Step	Life Scan/Johnson and Johnson	Glucose	Yes; Sure Step strips	Whole	5	Three AA 1.5 V alkaline batteries	0–500 mg/dl	89 × 61 × 20	107.7	Lieske *et al.* (2002), birds	10–35	10–90%
One Touch Ultra	Life Scan/Johnson and Johnson	Glucose	Yes; One Touch Ultra strips	Whole	1	One 3 V lithium battery	1.1–33.3	79 × 57 × 23	42	No	6–44	10–90%
Precision QID	Medisense	Glucose	Yes; MicroFlo Plus strips	Whole	3.5	Non-replaceable	1.1–33.3	97 × 48 × 15	39	No	4–30	Not available
Accusport Analyser	Boehringer Mannheim	Lactate	Yes; Lactate Test strips	Whole	10–20	Three 1.5 V AAA batteries	0.8–22	115 × 62 × 18.5	100	Wells and Pankhurst (1999), fish	5–35	10–90%
Accutrend	Roche Diagnostics	Lactate	Yes; Lactate Test strips	Whole	20–25	Three 1.5 V AAA batteries	0.8–22	115 × 62 × 18.5	100	No	5–35	10–90%
Lactate Pro	Arkray KDK	Lactate	Yes; Lactate Pro Strips	Whole	5	Two 3 V lithium batteries	0.8–23.3	83.8 × 55 × 14.5	50	No	10–40	20–80%
HemoCue	Hemocue 201+	Haemoglobin	Yes; meseauring cuvette	Non-specific	Non-specific	Four AA batteries	0–256 g/l	160 × 85 × 43	350	Clark *et al.* (2008)	Not available	Not available
BMS Hemoglo-binometer	BMS	Haemoglobin	Yes	Non-specific	Non specific	Two size ‘C’ batteries	4–20 g/dl	170 × 70 × 40	Not available	Iwama *et al.* (1995)	10–40	Not available
IQ128 Elite	IQ Scientific Instruments Inc.	pH	No	Non-specific	Non-specific	Two 3 V lithium batteries	pH 2–12	152.4 × 78.57 × 16.38	450	Brown *et al.* (2008), fish	5–40	Not available
WTW pH Meter pH330	Hoskin Scientific Ltd	pH	No	Non-specific	Non-specific	Four AA batteries	−2.00 to 19.99 pH units	172 × 80 × 37	300	No	−5 to 105	Not available
SevenGo Pro	Mettler Toledo	pH and ion	No	Non-specific	Non-specific	Four AA batteries	−2.00 to 19.99 pH units	220 × 90 × 45	325	No	0–40	0–85%
IRMA True Point	International Technidyne Corporation	Various; lactate, glucose, pH, variety of ions	Yes; various cartridges depending on parameters to be tested	Whole blood or plasma	125–500	One 7.2 V battery	Various ranges depending on parameters	292.1 × 211.3 × 127	2381	No	12–30	0–80% non-condensing
i-STAT	Abbott Point of Care	Various; lactate, glucose, pH, variety of ions	Yes; various cartridges depending on parameters to be tested	Non-specific	Non-specific	Two 9 V lithium batteries	Various ranges depending on parameters	209 × 64 × 52	520	No	16–30	0–90%
Ames Mini-lab	Miles Canada Inc.	Various	Not available	Not available	Not available	Not available	Not available	Not available	Not available	Iwama *et al.* (1995)	Not available	Not available

## Summary of taxon-specific validation and application studies

### Chondrichthyes

#### Validation studies

Only two studies were found that assessed the accuracy of POC devices with *Chondrichthyes* species. Both validation studies compared laboratory-based equipment with POC devices using linear regressions (Table 2). Gallagher *et al.* (2010) used the i-STAT analyser to measure acid–base parameters and/or a lone metabolite (lactate) in the whole blood of three different *Chondrichthyes* species. The i-STAT analyser was determined to be acceptable for the measurement of pH, partial pressure of oxygen (pO_2_) and carbon dioxide (pCO_2_) with temperature correction, as well as lactate, but given that derived correction factors varied by species and only a lone temperature point was examined, the authors cautioned against broad applicability across taxa and temperatures without further testing (Gallagher *et al.*, 2010). Awruch *et al.* (2011) determined that the use of the Lactate Pro was acceptable for measuring lactate, using a single species (i.e. *Galeorhinus galeus*), despite the fact that the device consistently overestimated lactate concentrations in whole blood relative to plasma. Further research is suggested into this lack of consistency between blood and plasma lactate values (Awruch *et al.*, 2011). While not a validation relative to traditional instrumentation, a third study found compatibility in side-by-side acid–base values between two POC instruments (i-STAT analyser vs. IRMA TruPoint analyser) when reading whole blood from minimally stressed chondrichthyans (Mandelman and Skomal, 2009). While more work is needed in parameters beyond acid–base, initial studies signify that POC devices can be acceptable tools for blood parameter readings in *Chondrichthyes*.

**Table 2:** The point-of-care (POC) device validation studies used in this study, grouped by class

Citation	Species	Temperature (°C)	POC device used	Standard method	Analyte measured	Comparison with POC reading	Acceptable comparison
Awruch *et al.* (2011)	Shark (*Galeorhinus galeus*)	Not available	Lactate Pro	Enzymatic kit/spectrophotometer	Lactate	Similar	Yes
Gallagher *et al.* (2010)	Sharks (*Carcharhinus plumbeus*, *Mustelus canis*)	25	i-STAT (CG4+)	Blood gas analyser (thermostatted)	pH	Similar	Yes
				Blood gas analyser (thermostatted)	pO_2_	Similar	Yes
				Blood gas analyser (thermostatted)	pCO_2_	Similar	Yes
				Laboratory lactate and glucose analyser	Lactate	Similar	Yes
Brown *et al.* (2008)	Bony fish (*Gadus morhua*)	Not available	Lactate Pro	Enzymatic kit/spectrophotometer	Lactate	Similar	Yes
Clark *et al.* (2008)	Bony fish (*Oncorhynchus nerka*, *Oncorhynchus tshawytscha*, *Thunnus orientalis*, *Scomber japonicus*)	Not available	HemoCue	Drabkin method	Haemoglobin	Higher	Somewhat
Cooke *et al.* (2008)	Bony fish (*Albula vulpes*)	21–25	i-STAT (E3+)	Laboratory chemistry analyser	Na^+^	Higher	Yes
					K^+^	Lower	Yes
					Cl^−^	Lower	Somewhat
				Centrifuge	Haematocrit	Variable	Yes
			Accu-Chek Advantage	Laboratory chemistry analyser	Glucose	Similar	Yes
DiMaggio *et al.* (2010)	Bony fish (*Fundulus seminolis*)	Not available	i-STAT (E3+)	Centrifuge	Haematocrit	Lower	No
				Flame photometer	Na^+^	Lower	No
				Flame photometer	K^+^	Lower	No
				Chloridometer	Cl^−^	Higher	No
Evans *et al.* (2003)	Bony fish (*Oreochromis niloticus*)	25–28	One Touch Ultra	Laboratory colorimetric method	Glucose	Lower	Yes
Harrenstien *et al.* (2005)	Bony fish (*Sebastes melanops*, *Sebastes mystinus*)	11.5	i-STAT (EC8+)	Laboratory chemistry analyser	Na^+^	Lower	Somewhat
				Laboratory chemistry analyser	K^+^	Similar	No
				Laboratory chemistry analyser	Cl^−^	Variable	No
				Laboratory chemistry analyser	BUN	Lower	Somewhat
				Laboratory chemistry analyser	Glucose	Similar	No
				Laboratory chemistry analyser	Haemoglobin	Lower	Somewhat
				Blood gas analyser	pH	Lower	Somewhat
				Blood gas analyser	pCO_2_	Higher	Somewhat
				Blood gas analyser	TCO_2_	Lower	No
				Blood gas analyser	HCO_3_^−^	Lower	No
				Blood gas analyser	Base excess	Lower	No
Iwama *et al.* (1995)	Bony fish (*Salmo salar*)	Not available	ExacTech	Laboratory assay kit	Glucose	Similar	Yes
			BMS Hemoglobinometer	Laboratory assay kit	Haemoglobin	Similar	Yes
			Ames minilab	Laboratory assay kit	Glucose, haemoglobin	Similar	Yes
Serra-Llinares *et al.* (2012)	Bony fish (*Gadus morhua*)	4	Lactate Pro	Reference values	Lactate	Similar	Yes
Wells and Pankhurst (1999)	Bony fish (*Oncorhynchus mykiss*)	14–40	Accuchek Advantage	Hexokinase method	Glucose	Lower	Somewhat
			Accusport	Enzymatic kit/spectrophotometer	Lactate	Lower	Somewhat
McCain *et al.* (2010)	Reptiles (*Pogona vitticeps*, *Tiliqua gigas*, *Geochelone platynota*, *Geochelone elegans*, *Boa constrictor*, *Pituophis melanoleucus*)	Not available	i-STAT (6+)	Laboratory chemistry analyser	Na^+^	Similar	Somewhat
					K^+^	Similar	Somewhat
					Cl^−^	Similar	Somewhat
					Glucose	Similar	Somewhat
Wolf *et al.* (2008)	Reptile (*Caretta caretta*, *Chelonia mydas*, *Lepidochelys kempii*)	18.9–27.2	i-STAT (EC8+)	Centrifuge	Haematocrit	Lower	Somewhat
				Laboratory chemistry analyser	Na^+^	Similar	Somewhat
				Laboratory chemistry analyser	K^+^	Similar	Somewhat
				Laboratory chemistry analyser	Cl^−^	Lower	Somewhat
				Laboratory chemistry analyser	Glucose	Lower	Somewhat
				Laboratory chemistry analyser	BUN	Higher	Somewhat
Lieske *et al.* (2002)	Bird (*Cerorhinca monocerata*)	Not available	Accu-Chek Advantage	Reagent strips	Glucose	Similar	Yes
			Precision QID	Reagent strips	Glucose	Similar	Yes
			Glucometer Elite	Reagent strips	Glucose	Similar	Yes
			Sure Step	Reagent strips	Glucose	Similar	Yes
Burdick *et al.* (2012)	Mammal (*Odocoileus virginianus*)	Not available	IQ Prestige Smart	Laboratory/portable chemistry analyser	Glucose	Variable	No
			Prestige Smart	Laboratory/portable chemistry analyser	Glucose	Variable	No
Hopper and Cray (2007)	Mammal (*Macaca fasicularis*)	Not available	i-STAT (EC8+)	Centrifuge	Haematocrit	Lower	Somewhat
				Laboratory chemistry analyser	Na^+^	Higher	Somewhat
				Laboratory chemistry analyser	K^+^	Similar	Somewhat
				Laboratory chemistry analyser	Cl^−^	Higher	Somewhat
				Laboratory chemistry analyser	BUN	Higher	Somewhat
				Laboratory chemistry analyser	Glucose	Lower	Somewhat
				Laboratory chemistry analyser	Haemoglobin	Higher	Somewhat
				Laboratory chemistry analyser	TCO_2_	Higher	Yes
Larsen *et al.* (2002)	Mammal (*Mirounga angustirostris*)	Not available	i-STAT (6+)	Laboratory chemistry analyser	Na^+^	Lower	No
				Laboratory chemistry analyser	K^+^	Similar	Yes
				Laboratory chemistry analyser	Cl^−^	Higher	Somewhat
				Laboratory chemistry analyser	BUN	Similar	Yes
				Laboratory chemistry analyser	Glucose	Lower	Somewhat
				Centrifuge	Haematocrit	Similar	Yes

Na^+^, sodium; K^+^, potassium; Cl^−^, chloride; BUN, blood urea nitrogen; TCO_2_, total carbon dioxide; pCO_2_, partial pressure of carbon dioxide; HCO_3_, bicarbonate; pO_2_, oxygen partial pressure. For each species and analyte, the POC and standard (control) device are presented together with the relative comparison between the two. Where possible, the relevant body/experimental temperatures are presented. The distilled opinions presented by the authors of each study are also presented, but case-by-case caveats are not reported here. Many papers with ‘acceptable’ comparisons argue the need for corrective calculations or find these devices acceptable for relative rather than absolute measurement of an analyte.

#### Application studies

As a result of their frequency of capture in commercial fisheries as bycatch and target species, many application studies of chondrichthyans have focused on the physiological consequences related to acute capture stress. The physiological effects of otter trawl capture in spiny dogfish was a reoccurring topic, presented by Mandelman and Farrington (2007a, b) as a duo of papers, which assessed pH, pO_2_ and pCO_2_ values in relationship to various aspects of capture (e.g. transport, captivity; Mandelman and Farrington, 2007b). Researchers have compared pH values generated with POC devices for *Chondrichthyes* captured by long-line (Mandelman and Skomal, 2009; Brooks *et al.*, 2011; Hyatt *et al.*, 2012), rod and reel (Brill *et al.*, 2008) and gillnet (Frick *et al.*, 2012; Hyatt *et al.*, 2012). Additional physiological parameters associated with aerial exposure and seasonality (Cicia *et al.*, 2012), tonic immobility (Brooks *et al.*, 2011) and reference ranges of wild and captive individuals (Naples *et al.*, 2012) have been studied. Most of these studies were conducted in the field, although four studies (Brill *et al.*, 2008; Brooks *et al.*, 2011; Cicia *et al.*, 2012; Naples *et al.*, 2012) were conducted fully or partly in a laboratory setting. A total of four POC devices were used to assess blood physiology in various *Chondrichthyes* species. The i-STAT analyser, a multi-parameter POC device, was the most commonly used device, while two single-parameter devices (Accu-chek glucometer and Lactate Pro lactate meter) were both used in more than one study. Numerous blood parameters were measured, with pH, lactate and pCO_2_ being the most commonly studied variables as indicators of acute stress.

### Teleost fish

#### Validation studies

Point-of-care devices have been used widely in teleost fishes. The accuracy of these devices has been validated by nine studies in teleosts, where POC devices were statistically compared with laboratory-based equipment (Table 2). The i-STAT analyser has been validated for use in bonefish (*Albula vulpes*; Cooke *et al.*, 2008) and Seminole killifish (*Fundulus seminoles*; DiMaggio *et al.*, 2010) as well as two species of rockfish (*Sebastes melanops* and *Sebastes mystinus*; Harrenstien *et al.*, 2005). Cooke *et al.* (2008) validated the i-STAT analyser for chloride (Cl^−^), sodium (Na^+^), potassium (K^+^) and haematocrit (Hct), and although the POC device and laboratory reference results deviated slightly, they concluded that relative differences could be determined accurately for bonefish. This differed from a study by DiMaggio *et al.* (2010), where the same blood parameters were assessed by the i-STAT, but the device was determined to be unsuitable for assessment in Seminole killifish, largely due to issues with blood clotting. In a third study on two species of rockfish by Harrenstien *et al.* (2005), the i-STAT was validated for pH, pCO_2_, Na^+^, urea nitrogen, Hct and haemoglobin (Hb); however, it was found to be unsuitable for glucose (due to a wide reference range), total CO_2_, bicarbonate (HCO_3_^−^) and K^+^ (due to unknown factors or device inconsistency), as well as Cl^−^ (and therefore anion gap; as these values were outside the measurable range of the device). The Ames Minilab and ExacTech glucose meter has also been validated for use in rainbow trout (*Oncorhynchus mykiss*) and Atlantic salmon for analysis of glucose and Hb but not erythrocyte number (Iwama *et al.*, 1995). Minilab erythrocyte number measurements were thought to have varied from laboratory reference values due to physiological differences in human and teleost red blood cells, because this device was originally calibrated for use on human samples (Iwama *et al.*, 1995). Generally, these studies suggest that the i-STAT, Minilab analyser and ExacTech glucose meter are useful in the measurement of blood parameters in teleost fish; however, species-specific validation is necessary for these devices because they were originally designed for use on mammals.

Glucose has also been validated for measurement by multiple versions of the Accu-chek, Freestyle Freedom Lite and the OneTouch Ultra glucose meter. Two other studies determined that glucose could be measured by the Accu-chek glucose meter in bonefish (Cooke *et al.*, 2008) and rainbow trout (Wells and Pankhurst, 1999). One further study validated the use of the OneTouch Ultra glucose meter in Nile tilapia (*Oreochromis niloticus*; Evans *et al.*, 2003). Overall, these studies suggested that relative rather than absolute values should be represented, because these devices tended to underestimate glucose values compared with laboratory reference values.

Two lactate POC devices, the Accusport and Lactate Pro lactate analysers, have been validated for use in teleost fishes. The Accusport (Wells and Pankhurst, 1999) lactate meter underestimated lactate levels compared with laboratory reference values in rainbow trout; similar to glucose, it was suggested that relative rather than absolute values should be represented. Alternatively, Brown *et al.* (2008) found that the Lactate Pro meter provided more accurate lactate values in Atlantic cod (*Gadus morhua*), when compared with laboratory reference values; however, resting lactate levels were below the detection limit of this meter. Serra-Llinares *et al.* (2012) has suggested that the Lactate Pro meter can effectively measure lactate levels from frozen plasma in Atlantic cod (*Gadus morhua*). These studies suggest that these two POC devices may provide reliable and useful lactate measurements.

Haemoglobin measurements by POC devices have been validated for the BMS Hemoglobinometer and the HemoCue haemoglobin analyser in four Salmonid and two Perciformes species. Iwama *et al.* (1995) found that the BMS Hemoglobinometer accurately measured Hb levels in two salmonid species; however, readings below 4 g/dl were not possible. Haemoglobin levels were generally overestimated by the HemoCue haemoglobin analyser in comparison to laboratory analysis (Drabkin's method) in two Salmonid and two Perciformes species; however, it was suggested that calibration equations could be applied to the data due to the systematic nature of the overestimation (Clark *et al.*, 2008). Overall, both POC devices may provide valuable Hb measurements, provided they are validated in the species of interest prior to application.

#### Application studies

Teleosts represent an important group of vertebrates in fisheries and aquaculture; as such, there is a demand for understanding the physiological impacts of these practices on fish. Point-of-care devices provide a convenient method to assess the effects of commercial and recreational fishing on a number of fish species. The effects of catch-and-release angling and barotrauma have been assessed mainly using glucose and lactate levels as indicators of stress, as well as Hct and Hb in smallmouth and largemouth bass (*Micropterus salmonides*; Gravel and Cooke, 2008; Hanson *et al.*, 2008; White *et al.*, 2008; Nguyen *et al.*, 2009; Thompson *et al.*, 2012), muskellunge (*Esox masquinongy*; Landsman *et al.*, 2011), northern pike (*Esox lucius*; Arlinghaus *et al.*, 2009), great barracuda (*Sphyraena barracuda*; O'Toole *et al.*, 2010), snapper (*Pagrus auratus*; Wells and Dunphy, 2009) and bonefish (Suski *et al.*, 2007). Henry *et al.* (2009) quantified the effects of fishing lure retention on smallmouth bass (glucose and lactate), while Roth and Rotabakk (2012) assessed the consequences of commercial and recreational fisheries in saithe (*Pollachius viren*s; multiple parameters).

Field and laboratory-based experiments as well as aquaculture facility practices generally require fish handling. As such, the physiological effects of fish manipulation have been assessed in a number of studies using POC devices. Fish capture, transportation, holding and sedation are some of the common practices involved when researching teleosts. Using bonefish, Murchie *et al.* (2009) assessed the effect of capture, transport and long-term holding, while Cooke *et al.* (2008) evaluated the effect of different capture techniques to assess post-capture stress on a number of blood physiology parameters. Moran *et al.* (2008) assessed the effect of hypercapnic conditions associated with transportation on glucose and lactate levels in yellowtail kingfish (*Seriola lalandi*). Further studies investigated the physiological effects of sedation on fish. The effects of electrosedation on glucose and lactate levels were examined in grass carp (*Ctenopharyngodon idella*; Bowzer et al., 2012), hybrid striped bass (*Morone chrysops* × *Morone saxatilis*; Trushenski and Bowker, 2012; Trushenski *et al.*, 2012a) and largemouth bass (Trushenski *et al.*, 2012b). Anaesthetics used prior to harvest in channel catfish (Bosworth *et al.*, 2007) have been assessed for various parameters; in addition, the effects of the overall anaesthetic efficiency and how this affects the quality of RNA extracted (Olsvik *et al.*, 2007) have been studied. The post-mortem effects of carbon monoxide (Bjørlykke *et al.*, 2011) and pre-slaughter live-chilling sedation effects (Foss *et al.*, 2012) have been examined in Atlantic salmon. Point-of-care devices have also been used to measure glucose, potassium and sodium in Atlantic salmon fillets to determine retention of the synthetic antioxidant, butylated hydroxyanisole (Petri *et al.*, 2008).

In addition to capture and handling, teleosts are often exposed to a number of other abiotic and biotic stressors. For example, using glucose and/or lactate levels, Bjørn *et al.* (2001) studied infection of brown trout (*Salmo trutta*) by salmon lice, Evans *et al.* (2003) studied sub-lethal dissolved oxygen stress and susceptibility to *Streptococcus algalactiae* in Nile tilapia, and Breau *et al.* (2011) studied the effects of increased water temperature on Atlantic salmon. Effects of captive rearing have been studied by looking at the effects of water reuse and stock density on growth rate in juvenile cod (*Gadus morhua*; Foss *et al.*, 2006) and the interactive effects of ammonia and oxygen on the growth and physiology of juvenile Atlantic cod (Remen *et al.*, 2008). Several additional studies have examined effects of anthropogenically induced stressors, mainly on glucose and lactate levels, but also other blood parameters, including blood gas and ion levels, Hct and Hb levels. The effects of confinement in rainbow trout (Wells and Pankhurst, 1999), as well as pre-slaughter stress and stress assessment in aquaculture facilities in Atlantic cod (Hultmann *et al.*, 2012), have been examined. Surgical techniques and recovery have been examined, specifically focusing on hepatic portal vein cannulation technique in Atlantic salmon (Eliason *et al.*, 2007). In addition, the effect of squeezing to simulate gill net damage in rainbow trout (Kojima *et al.*, 2004) and stress associated with dam-related changes in river flow in mountain whitefish (*Prosopium williamsoni*; Taylor *et al.*, 2012) have been assessed. Finally, the effects of various toxicants and pollutants on blood parameters have been examined in a cichlid species (*Cichlasoma dimerus*; Da Cuña *et al.*, 2011), two salmonid species (Meland *et al.*, 2010; Olsvik *et al.*, 2010) and the round goby (*Neogobius melanostomus*; Marenetette *et al.*, 2012).

The manipulation of teleost physiology is also of particular relevance to investigators interested in understanding the intrinsic principles of physiological processes, as well as the result of these manipulations on physiological processes. Point-of-care devices provide a means to examine such physiological end-points easily. Dey *et al.* (2010) examined the effect of altering cortisol and androgen levels on glucose levels during the parental care period of smallmouth bass. In addition, Herbert *et al.* (2002) measured glucose and lactate levels during strenuous exercise in 14 species of tropical reef fish. Laporte and Trushenski (2012) studied the effect of manipulating the composition of aquafeeds on glucose and/or lactate levels in hybrid striped bass. Turbot (*Scophthalmus maximus*) were used to determine the interaction effects of oxygen saturation on growth and blood physiology (Foss *et al.*, 2007).

Overall, 10 POC devices have been used in applied studies to assess blood physiology parameters in teleost fish; however, not all devices have been validated for use in teleosts. The Accu-chek glucose meter and Lactate Pro were the most widely used POC devices, followed by the i-STAT, Freestyle blood glucose meter, Accutrend and Accusport lactate meters. The use of these POC devices in teleost fish across a wide range of applied studies demonstrates not only their usefulness in field and laboratory-based physiology, but also the need for further species-specific validation of these tools.

### Reptiles

#### Validation studies

Two studies were conducted to validate the use of the i-STAT analyser for various blood parameters on reptiles (Table 2). McCain *et al.* (2010) concluded that whole-blood readings for Cl^−^, glucose, K^+^ and Na^+^ using the i-STAT analyser in various reptiles were not accurate compared with laboratory measurements. Despite its variation from laboratory-based results, McCain *et al.* (2010) suggested that as a result of consistently biased values, the i-STAT could provide clinical utility if analyser-specific reference intervals were set. Wolf *et al.* (2008) compared the i-STAT analyser with four other analysers (two laboratory diagnostic methods and two table-top analysers) using whole blood from various sea turtle species [loggerhead turtles (*Caretta caretta*), green turtles (*Chelonia mydas*) and Kemp's ridley turtles (*Lepidochelys kempii*)] for Na^+^, K^+^, Cl^−^, glucose, blood urea nitrogen (BUN) and Hct. In most cases, i-STAT readings disagreed with other analysers, except for BUN, which the authors proposed was a result of differences in the mechanisms used to measure the analytes. Given that the use of POC analysers has generated mixed results in reptiles in general, additional research to validate various POC devices in this group is warranted.

#### Application studies

Five studies have used POC devices to measure blood parameters, such as acid–base properties, in reptiles. The i-STAT analyser was the most frequently used device, with three studies using this tool to assess the health and wellbeing of various sea turtle species. Sea turtles are frequently encountered as bycatch in marine fisheries, and two studies assessed the physiological effects of different capture and handling techniques. Harms *et al.* (2003) examined the differential effects of trawl vs. pound net in loggerhead sea turtles and Innis *et al.* (2010) evaluated the physiological status and health of leatherback sea turtles directly captured by hoop net or incidentally entangled in fixed (i.e. stationary) fishing gears. Anderson *et al.* (2011) also measured blood parameters in green turtles that were cold stunned or hypothermic. In addition, bycatch-related blood physiology was assessed in freshwater fisheries using the Lactate Pro and IQ128 Elite pH meter to evaluate the physiological response to various types of bycatch-reduction devices, using painted turtles (*Chrysemys picta*; Larocque *et al.*, 2012) as well as eastern musk (*Sternotherus odoratus*) and northern map turtles (*Graptemys geographica*; Stoot *et al.*, 2013). Given the recent increase in research effort, the use of POC analysers is becoming a more common mode to evaluate stress and health in both marine and freshwater reptiles.

### Birds

#### Validation studies

Based on our search criteria, only one study has validated the use of POC devices for avian species (Table 2). Lieske *et al.* (2002) used adult rhinoceros auklets (*Cerorhinca monocerata*) to assess the accuracy of four POC devices (Accu-chek Advantage, Bayer Glucometer Elite, Precision QID and Sure Step). Based on mean difference and regression models, all devices were deemed reliable and potentially useful for screening in the field, although the four hand-held devices underestimated blood glucose of rhinoceros auklets by an average of 33% compared with reference values (Lieske *et al.*, 2002). Based on ease of use, comparative accuracy, test time, cost and blood volume requirement, the Accu-chek Advantage and Precision QID monitors were the most appropriate POC devices for this avian model, and future research is suggested with known hypoglycaemic avian blood as well as blood from different species to assess overall utility of the devices (Lieske *et al.*, 2002).

#### Application

Two POC devices have been used in practical applications to measure glucose and numerous other physiological parameters in avian species. Researchers have used the Bayer Glucometer Elite device in the field and laboratory to assess the possible effect of nectar consumption on plasma glucose in various passerine species, including warblers (Cecere *et al.*, 2011), and to determine whether plasma glucose levels were based on circadian rhythm or temperature (Downs *et al.*, 2010). Although not validated for avian species, the i-STAT analyser has been used to analyse blood acid–base, ionic and haematological properties to provide reference data for non-anaesthetized Amazon parrots (*Amazona aestiva*; Paula *et al.*, 2008).

### Mammals

#### Validation

Our search discovered three studies that have assessed the accuracy of POC devices for use in non-domesticated mammals (Table 2). All but one validation study compared the i-STAT analyser with laboratory-based equipment to measure various blood parameters in three different mammalian species. The i-STAT analyser was determined to be acceptable for the measurement of glucose, BUN, Na^+^, K^+^ and total CO_2_ in cynomolgus macaques (*Macaca fasicularis*; Hopper and Cray, 2007) and glucose, BUN, Na^+^, K^+^, Cl^−^ and Hct in elephant seals (*Mirounga angustirostris*; Larsen *et al.*, 2002). In addition, Burdick *et al.* (2012) compared laboratory-based equipment (Hitachi 917 chemistry analyser) with the IQ Prestige Smart System Handheld Glucometer and the Prestige Smart System Glucometer for measurement of blood glucose levels in juvenile white-tailed deer (*Odocoileus virginianus*). Agreement between laboratory-based equipment and the two POC glucometers was poor, thus driving the conclusion that these two POC devices were not appropriate for measurement of blood glucose concentrations in this species.

#### Applications

Two POC devices, the i-STAT analyser and Accutrend lactate meter, were used to assess blood physiology in various non-domesticated mammal species. Of the six application studies completed to date, all six used the i-STAT, with pH and blood gases being the most commonly measured variables. Application studies examined the physiological effects related to invasive capture techniques in white-tailed deer (Boesch *et al.*, 2011), anaesthetics in polar bears (*Ursus maritimus*; Cattet *et al.*, 2003), immobilization in Baird's tapirs (*Tapirus bairdii*; Feorster *et al.*, 2000) and manual restraint as opposed to anaesthetic in the Arabian oryx (*Oryx leucoryx*; Kilgallon *et al.*, 2008). Application studies also examined the physiological effects related to sleep apnoea in Northern elephant seals (*Mirounga angustirostris*; Stockard *et al.*, 2007), as well as an assessment and post-release monitoring of mass-stranded dolphins (Sampson *et al.*, 2012).

## Limitations across taxonomic groups

### General limitations

Of the devices used in this data set, two broad categories of POC devices can be designated: the widely applicable multi-analyte devices (e.g. i-STAT analyser) and the specialized single-analyte devices (e.g. Lactate Pro). The i-STAT analyser is beneficial in that it can be used to test a variety of analytes, but it requires a certain amount of user knowledge and training in order to operate the machine settings, to dispense the sample into the cartridge appropriately and to avoid cartridge errors. These devices require a working knowledge of the device as well as the physiology of the organism but are ideal for obtaining multiple physiological measures from a single sample. Conversely, single-analyte devices, such as the Lactate Pro, are more specialized in scope, function on a limited number of analytes and operate on the submitted blood sample without further input or setting configuration. These devices are typically small, easy to use, relatively inexpensive and ideal if only one blood variable measurement is required.

### Physiological limitations

As a result of their origins in medical sciences, POC devices were designed to measure blood parameters of homeothermic mammals, particularly humans. These devices rely on individuals with a body temperature of ∼37°C and non-nucleated blood cells, thus the ability of POC devices to be applied to a broader variety of taxa will have challenges. Consequently, body temperature was a reoccurring caveat across studies of ectotherms using these devices, especially in relationship to temperature-sensitive blood acid–base measurements (Harrenstien *et al.*, 2005; Gallagher *et al.*, 2010; Sampson *et al.*, 2012). Indeed, several studies concluded that there is a need for species-specific temperature corrections for these parameters and emphasized that extrapolation of published temperature corrections from one species to another should be done with caution [e.g. *Chondrichthyes* (Mandelman and Skomal, 2009; Cicia *et al.*, 2012), teleost fish (Olsvik *et al.*, 2010; Foss *et al.*, 2012), reptiles (Harms *et al.*, 2003; Anderson *et al.*, 2011) and birds (Paula *et al.*, 2008)].

Inaccurate readings can be the result of incorrect measurements. Many devices rely on pre-set ratios to calculate parameters and have species-specific correction values, which differ among species and taxa. For example, the i-STAT analyser was not used to measure base excess and saturated arterial oxygen in sea turtles due to its use of human-specific conversion factors (Harms *et al.*, 2003). Along the same lines, the internal temperature calibration capability for pH and blood gases on the i-STAT analyser is based on mammalian conversion factors, and may thus compromise accuracy of values of those parameters when the conversion tool is employed for ectotherms, such as fish (Mandelman and Skomal, 2009). Glucose monitors have similar issues, because they are designed to measure whole-blood samples but use a correction factor to convert results to plasma glucose concentrations (Kuwa *et al.*, 2001). These devices use human plasma-to-whole-blood corrective values, which can underestimate glucose values in birds (Lieske *et al.*, 2002; Acierno *et al.*, 2008). In addition, this trend for underestimation is also seen in POC lactate measurements in teleost fish (e.g. Wells and Pankhurst, 1999; Venn Beecham *et al.*, 2006).

### Whole blood vs. plasma and point-of-care value range restrictions

The input medium is important to consider when using POC devices, where whole blood and plasma are the two most prevalent media used. Whole blood is unmodified (with the potential exception of the addition of an anticoagulant) and is therefore ideal for field studies where processing and storage may be difficult or impossible. Plasma is extracellular fluid retrieved by spinning down whole blood by centrifugation to remove haematocytes. Plasma is often the preferred medium for laboratory studies owing to its higher stability for storage compared with whole blood (Thrall *et al.*, 2012); however, the need for centrifugation can limit feasibility in the field. Although urine may be analysed by POC devices for some analytes, it is not as widely used. For example, urine cannot be tested reliably using the i-STAT analyser (Erickson and Wilding, 1993). Serum can also be used as an input medium, but it has certain advantages and disadvantages similar to plasma (Thrall *et al.*, 2012).

In the studies investigated here, the vast majority assessed whole blood when using a POC device, while some used plasma, particularly those in controlled environments where plasma was often stored and analysed at a later date. Validation studies that measured the difference between whole blood and plasma analyte levels found that the analyte value differed significantly but in a predictable manner, with plasma values typically being higher (Iwama *et al.*, 1995). The persistent differences between plasma- and whole-blood-derived values allow for relative but not direct comparisons, which limits inference across studies (Larsen *et al.*, 2002; Cooke *et al.*, 2008).

**Figure 1:**
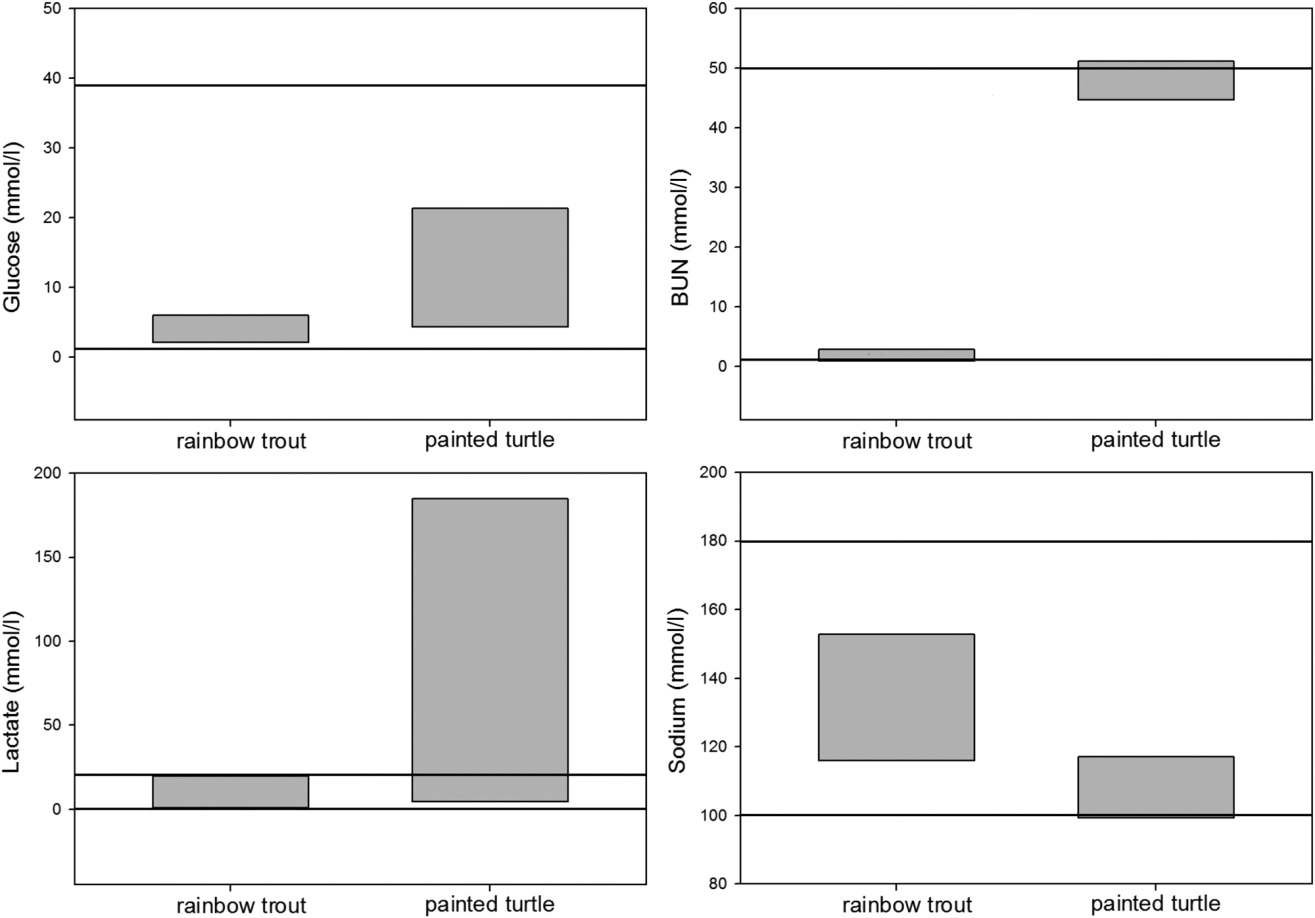
Whole blood values for select analytes on representative commonly studied species, the rainbow trout (*Oncorhynchus mykiss*) and the painted turtle (*Chrysemys picta*). Box plots represent the maximal ranges from the literature and horizontal lines represent the maximal reportable range of the most commonly used point-of-care device in the present study, the i-STAT analyser.

As these devices are designed for human blood parameter ranges, their tolerances can often be too restrictive for other taxa and result in error readings [e.g. *Chondrichthyes* (Awruch *et al.*, 2011; Hyatt *et al.*, 2012; Naples *et al.*, 2012), teleost fish (Harrenstien *et al.*, 2005; Brown *et al.*, 2008), reptiles (Larocque *et al.*, 2012) and birds (Lieske *et al.*, 2002); Fig. 1). For example, many studies that used the i-STAT analyser to assess blood physiology in teleost fish and *Chondrichthyes* have reported out-of-range readings for various parameters, including Na^+^ (Olsvik *et al.*, 2007; Suski *et al.*, 2007; Brill *et al.*, 2008) and Cl^−^ (Harrenstien *et al.*, 2005; Brill *et al.*, 2008; DiMaggio *et al.*, 2010). In situations when limits are too narrow, dilutions can be used to obtain measurable readings (Suski *et al.*, 2007; Brill *et al.*, 2008); however, this can only be achieved with plasma, creating a problem for any cartridge type (e.g. the EC8_+_ on the i-STAT) on a multi-analyte device that includes both out-of-range analytes as well as those specific to whole blood for a given species (e.g. certain acid–base and haemotogical parameters). In such instances, the user is unable to obtain value readings for all analytes for that cartridge and must prioritize whether to run the cartridge on whole blood or on extracted and diluted plasma, depending on which analyte(s) is less valuable to lose.

### Field and environmental limitations

**Figure 2:**
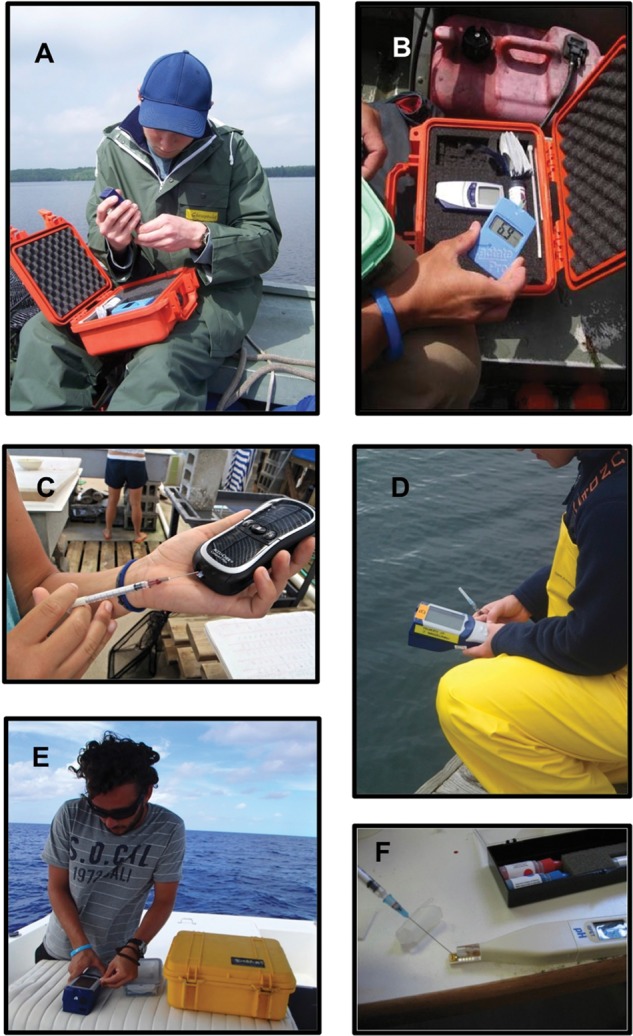
Point-of-care (POC) devices can be used in the field in a variety of situations. (**A**) Point-of-care glucose meter being set up on a boat (photograph by Lisa Thompson). (**B**) Protective cases, similar to the one shown, are useful for ensuring that POC devices are not damaged by the elements (photograph by Lisa Thompson). (**C**) Point-of-care devices, such as this glucose meter, can be used in laboratory trials to obtain immediate results on the condition of the individual (photograph by Petra Szekeres). (**D**) Some POC devices, such as the i-STAT, can obtain multiple blood parameters to be measured from one sample (photograph by John Mandelman). (**E**) Point-of-care i-STAT device in use on a boat (photograph by John Mandelman). (**F**) Point-of-care pH meter in use to examine blood pH in freshwater turtles (photograph by Sarah Larocque).

When conducting fieldwork, researchers may encounter several different environmental conditions that have the potential to interfere with POC device use (Fig. 2). Waterproofing becomes necessary when working with species in aquatic settings to protect devices from potential water damage. Small amounts of water (especially saltwater) within cartridge ports and battery compartments of POC devices can compromise the condition of the unit and damage it. Waterproof cases protect devices from water and general damage during transport, although depending on case size, portability can be reduced. In addition, these cases protect the device only while in transit, and care must be taken during device use to avoid potential submersion. Future steps to develop POC devices that are waterproof and more durable would reduce the need for bulky cases.

The effects of temperature and atmospheric humidity on the usability and accuracy of POC devices are further potential issues when working in the field. Studies conducted on the use of POC devices in disaster-relief situations have concluded that temperature and humidity can affect glucose test strips, which alters the readings of various glucose monitors (Louie *et al.*, 2000; Nichols, 2011). Many POC devices and their associated cartridges have environmental range limits in which they function optimally, and deviations outside of this range can produce inaccurate readings (Sampson *et al.*, 2012). Functional minima and maxima (e.g. i-STAT analyser has a functional range of 16–30°C; Lactate Pro cannot function properly above 40°C) limit the possible field season in some climates and preclude sampling altogether in some areas (e.g. polar and desert regions). Users are often forced to carry a cooling or heating mechanism to ensure that the POC device is kept within the usable thermal range. In addition, many POC devices use a cartridge or strip that requires storage at specific temperatures (e.g. i-STAT cartridges require refrigeration before use and can be kept at room temperature for only 2 weeks following removal). Furthermore, these devices are affected by extreme humidity conditions, such that most devices operate between 10 and 90% relative humidity (e.g. Lactate Pro and i-STAT operate in the range 20–80 and 0–90% relative humidity, respectively). Overall, field settings can make storage and maintenance of POC devices in varying temperatures and humidities difficult. As such, modifications of POC devices to incorporate a broader thermal and humidity tolerance is needed before these devices can be considered fully effective for studies of species inhabiting a wide range of environments.

## Conclusions and future directions

Overall, most POC devices have been found to be suitable alternatives to traditional laboratory-based devices in conservation physiology studies, although they should be used with caution. With continuing technological developments, such devices have the potential to be used more widely in field physiology studies on a variety of taxa. However, the popularity of such devices will depend on technological advances in the usability and reliability of POC devices in the field. Environmental conditions (e.g. temperature and humidity) affect the usability and accuracy of POC devices; as such, POC devices need to be functional broadly in field environments and require improvements or modifications to overcome their functional limitations (temperature range, humidity, storage requirements, maintenance etc.). Technological advances will increase the applicability of POC devices to a variety of species living in different field conditions or in wide demographic ranges. This will ensure that extreme or fluctuating environmental field conditions do not interfere with the use of POC devices by damaging the devices or affecting the accuracy of the results. Partnerships and collaborations between field biologists and manufacturing companies can help to formulate such technological improvements.

In addition to technological advancements of POC devices, there is an increased need for validation studies on non-human and non-domesticated species to confirm the accuracy and reliability of these devices, particularly given the lack of universality in the calibrations of POC devices among species evaluated to date. Therefore, precise calibration and species-specific validation of POC devices are necessary prior to application in a broader range of species (Lieske *et al.*, 2002; DiMaggio *et al.*, 2010; Gallagher *et al.*, 2010). Validation studies should address taxonomic and thermal effects on device precision and accuracy (Cicia *et al.*, 2012). In particular, efforts should be made to garner larger sample sizes to compare with current reference values (Naples *et al.*, 2012). Optimally, a calibration can be established across the broadest possible range of values for a given analyte, in the event of a lack of equivalence or linearity across the full spectrum of readable or biologically/clinically relevant values. Furthermore, biases can occur among taxa, where validation studies tend to be carried out in species or groups that are easy to study or are of economic importance (e.g. Atlantic salmon). The diversity among taxa in terms of habitat ecology and blood physiology points to the need for further investigation into a broader range of organisms, especially non-mammalian taxa. Focusing on representative models within each taxa, validating commonly used devices and assessing blood parameters in natural, free-living animals would be a good starting point. Validations of these devices on ectothermic species should be of high importance due to their physiological differences from endothermic species. In addition, developing a standardized protocol to validate devices, within the taxon level, would be beneficial and aid with the standardization of validations.

With the growing use of POC devices in field-based conservation physiology studies, there is a push towards the streamlined approach that is provided by multi-analyte devices (e.g. i-STAT). The ability to measure multiple blood parameters from a single sample by a single device is ideal for field-based studies, where extensive sample analysis is often difficult. Currently, the majority of the POC devices (e.g. LactatePro and various glucometers) measure one parameter exclusively, which can not only increase the equipment load for field biologists but also the amount of sample required. The development of more multi-analyte devices that are able to measure multiple blood parameters efficiently and accurately across a variety of taxa will increase the use of these devices by field physiologists.

In addition to the current POC devices available, there is the potential to develop devices that could measure blood parameters beyond those that are currently possible, again allowing conservation physiologists the flexibility of on-site sample analysis. Point-of-care devices that would enable direct measurement of primary in addition to secondary stress analytes would allow remote determination of levels now attainable only via more time-consuming processes in the laboratory. The ability to obtain POC measurements of glucocorticoids or other steroids (such as reproductive hormones), among other biomarkers, would be highly valuable for field-based conservation physiology studies.

In conclusion, the use of hand-held and portable POC devices is appealing for field-based conservation physiology studies because they rapidly provide on-site results without the need for sample storage. Overall, there is great potential for the use of POC devices to advance the field of conservation physiology, but continued progress is needed in the areas discussed to increase both the utility of these devices across environments and taxonomic groups and their capacity to obtain additional information on stress levels and health of animals in remote settings. Also needed is a more thorough appreciation of the various limitations associated with POC devices and recognition that although they provide rapid information, they do not replace traditional analytical methods.
